# 18F-FDG PET/CT image findings of a dog with adrenocortical carcinoma

**DOI:** 10.1186/s12917-021-03102-6

**Published:** 2022-01-03

**Authors:** Dohee Lee, Taesik Yun, Yoonhoi Koo, Yeon Chae, Dongwoo Chang, Mhan-Pyo Yang, Byeong-Teck Kang, Hakhyun Kim

**Affiliations:** 1 Laboratory of Veterinary Internal Medicine, College of Veterinary Medicine, Chungbuk National University, Cheongju, Chungbuk 28644 South Korea; 2 Department of Veterinary Imaging, Veterinary Teaching Hospital, College of Veterinary Medicine, Cheongju, Chungbuk 28644 South Korea

**Keywords:** Adrenocortical carcinoma, Adrenal tumor, Canine, Fluorodeoxyglucose, Positron emission tomography

## Abstract

**Background:**

In human medicine, 18F-fluorodeoxyglucose (FDG) positron emission tomography (PET) has been used to differentiate between benign and malignant adrenal tumors and to identify metastases. However, canine adrenocortical carcinomas identified by 18F-FDG PET/computed tomography (CT) have not been reported.

**Case presentation:**

A 13-year-old, castrated male, Cocker Spaniel dog with severe systolic hypertension exhibited an adrenal mass approximately 3.6 cm in diameter on ultrasonography. There was no evidence of pulmonary metastasis or vascular invasion on thoracic radiography and abdominal ultrasonography, respectively. 18F-FDG PET/CT was performed to identify the characteristics of the adrenal mass and the state of metastasis. One hour after injection of 5.46 MBq/kg 18F-FDG intravenously, the peripheral region of the adrenal mass visually revealed an increased 18F-FDG uptake, which was higher than that of the liver, and the central region of the mass exhibited necrosis. The maximal standardized uptake value (SUV) of the adrenal mass was 3.24; and relative SUV, calculated by dividing the maximal SUV of the adrenal tumor by the mean SUV of the normal liver, was 5.23. Adrenocortical carcinoma was tentatively diagnosed and surgical adrenalectomy was performed. Histopathologic examination of the resected adrenal mass revealed the characteristics of an adrenocortical carcinoma. After adrenalectomy, systolic blood pressure reduced to below 150 mmHg without any medication.

**Conclusion:**

This is the first case report of 18F-FDG PET/CT findings in a dog with suspected adrenocortical carcinoma and may provide valuable diagnostic information for adrenocortical carcinoma in dogs.

## Background

Primary adrenal tumor is an uncommon, but well-recognized condition in veterinary medicine [1, 2]. While urine catecholamine testing can aid the diagnosis of some pheochromocytomas, surgical resection and histopathologic assessment remains the definitive method for adrenal tumor diagnosis. Regardless of the tumor origin, adrenalectomy is necessary for a definitive diagnosis and treatment in dogs with primary adrenal tumors. However, the overall perioperative mortality rate for dogs undergoing adrenalectomy for all adrenal tumors is approximately 20% [3, 4], and much higher mortality rate (48%) is reported in dogs with pheochromocytoma without a pre-operative treatment of phenoxybenzamine [5]. Fortunately, pre-operative administration of phenoxybenzamine (an α-adrenergic antagonist) is reported to reduce the perioperative mortality rate in canine pheochromocytoma to 18% [5]. Therefore, clinicians attempt to identify origin (adrenal cortex or medulla), functionality (functional or non-functional), and biological nature (benign or malignant) of the adrenal mass based on clinical signs, hormonal analyses, and imaging modalities before adrenalectomy.

In human medicine, 18F-fluorodeoxyglucose (FDG) positron emission tomography (PET) imaging presents metabolic information to differentiate between benign and malignant tumors [6, 7] and to identify metastases in various tumors; glucose metabolism is increased in malignant lesions [8, 9]. Reportedly, the sensitivity and specificity of 18F-FDG PET in differentiating benign from malignant adrenal tumors are 97 and 91%, respectively [9], and it exhibits a higher sensitivity than computed tomography (CT) in the detection of metastases of adrenal tumor [10].

To our knowledge, there are no previous reports of canine adrenal tumors identified by 18F-FDG PET/CT, and there has been only one report of two dogs with pheochromocytoma visualized by PET/CT using p-[18F] fluorobenzylguanidine [11]. Therefore, this case report is the first to demonstrate the use of 18F-FDG PET/CT findings in a clinical case of a dog histologically diagnosed with adrenocortical carcinoma.

## Case presentation

A 13-year-old, castrated, male Cocker Spaniel dog weighing 10.1 kg was referred for persistent hypertension despite the administration of anti-hypertensive drugs. Before referral, the dog was treated with oral amlodipine 0.2 mg/kg/q24h and hydralazine 0.7 mg/kg/q12h for 5 weeks. At presentation, severe hypertension of approximately 300 mmHg, measured by the Doppler method, was identified. Complete blood count showed lymphopenia (377/μL; reference range 1050–5100/μL). Biochemical and electrolyte findings included mildly increased alkaline phosphatase activity (275 IU/L; reference range 29–97 IU/L), mild hypernatremia (156 mmol/L; reference range 141–152 mmol/L), and mild hyperchloremia (118 mmol/L; reference range 105–115 mmol/L).

Thoracic and abdominal radiographs revealed no remarkable abnormality. Abdominal ultrasonography revealed a heterogeneous hypoechoic adrenal mass, approximately 3.6 cm at its maximum diameter, cranioventral to the right kidney, without any evidence of vascular invasion or metastasis. The left adrenal gland was not considered atrophied because its maximal thickness was 0.61 cm, which is more than the normal observed thickness of 0.5 cm [12].

To determine whether the adrenal mass was functional or non-functional, urinary normetanephrine-to-creatinine ratio and serum concentrations of cortisol, 17-hydroxyprogesterone (OHP), estradiol, and progesterone before and after the intravenous administration of 250 μg synthetic adrenocorticotropic hormone (ACTH) were measured. The urine normetanephrine-to-creatinine ratio was 247.56, which could not completely exclude the possibility of pheochromocytoma or confirm it [13]. Concentrations of 17-OHP, estradiol, and progesterone were found to be markedly increased both prior to and after the ACTH stimulation test; basal 17-OHP = 1.32 ng/mL [reference interval (RI), < 0.1 ng/mL], post-stimulation 17-OHP = 6.87 ng/mL (RI 0.4–1.2 ng/mL), basal estradiol = 78.9 pg/mL (RI 28–63 pg/mL), post-stimulation estradiol = 93.2 pg/mL (RI 30–69 pg/mL), basal progesterone = 1.04 ng/mL (RI < 0.1 ng/mL), and post-stimulation progesterone = 8.47 ng/mL (RI 0.4–1.2 ng/mL). The basal cortisol concentration was within the reference range (2.09 μg/dL; RI, 1.0–6.0 μg/dL), but post-ACTH stimulation cortisol concentration was below the reference range (6.26 μg/dL; RI, 7–17 μg/dL) [14]. The ACTH stimulation test has poor sensitivity and is considered diagnostically inferior to the low dose dexamethasone suppression test (LDDST) in dogs with adrenal tumor-dependent hyperadrenocorticism. The LDDST was suggested, but the owners declined. These results were not sufficient to differentiate between pheochromocytoma and adrenocortical tumors.

**Fig. 1:**
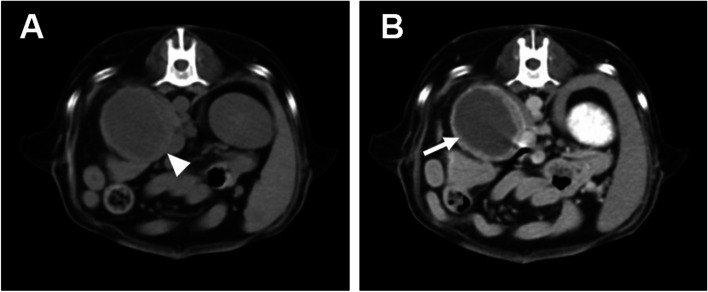
Computed tomography (CT) images of right adrenal mass of the dog. **a** On pre-contrast CT image, the adrenal mass (an arrow head) showing a hyperattenuated peripheral region and homogeneously hypoattenuated central region. The size of the adrenal mass was 4.3 × 6.8 cm in diameter. **b** On post-contrast CT image, contrast enhancement (arrow) occurred from the peripheral region of the mass

**Fig. 2:**
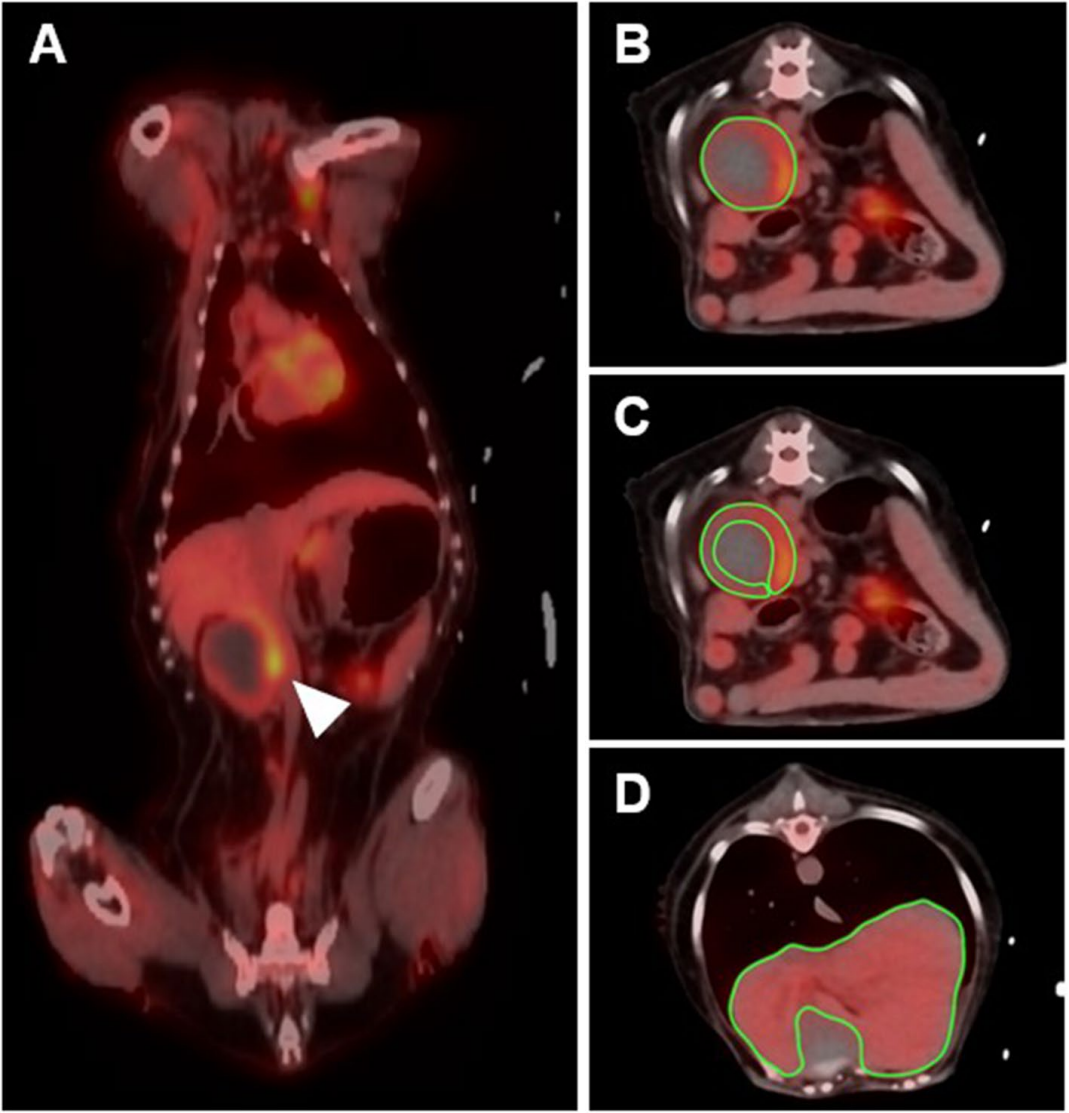
Fused positron emission tomography/computed tomography images using 18F-fluorodeoxyglucose in a dog with adrenocortical carcinoma. **a** Increased 18F-fluorodeoxyglucose uptake of the peripheral region of the right adrenal mass (an arrow head) was observed and central region of the mass revealed necrosis in dorsal plane, **b** Region of the interest (ROI) was drawn in transverse plane for adrenal mass. Maximal standardized uptake value (SUV) is 3.24 and mean SUV is 0.36. **c** ROI was drawn in transeverse plane for peripheral region of the adrenal mass. Mean SUV is 0.79. **d** ROI was drawn in transeverse plane for liver. Mean SUV is 0.62. Relative SUV (adrenal SUVmax/liver SUVmean = 3.24/0.62) is 5.23

Thus, CT and 18F-FDG PET/CT scan of the whole body were performed under isoflurane inhalation anesthesia to identify characteristics of the right adrenal mass and state of metastasis. CT images were obtained with a four-row multi-detector CT scanner with 100 mAs and 120 kVp in 1.25 mm slice thickness. Post-contrast CT scans were performed 2 min after intravenous injection of 880 mgI/kg iohexol. A right adrenal mass was detected on the CT, with a size was 4.3 × 6.8 cm diameter (Fig. 1A). The peripheral region of the mass was hyperattenuated and central region was homogenously hypoattenuated. Post-contrast images showed contrast enhancement of the peripheral region of the mass (Fig. 1B). PET/CT using 18F-FDG was performed 1 h after the injection of 5.46 MBq/kg 18F-FDG intravenously [15]. The peripheral region of the adrenal mass visually revealed an increased 18F-FDG uptake, which was higher than that of the liver and the central region of the mass exhibited an absent 18F-FDG uptake (Fig. 2A). The PET image was analyzed using the commercial program (OsiriX MD v10.0, Pixmeo, Bernex, Switzerland). The regions of interest (ROIs) were drawn manually on the PET/CT fusion images and were transferred to a standardized uptake value (SUV) as follows: SUV = average tissue concentration of 18F-FDG (MBq/mL)/injected dose (MBq) per body weight (g). The maximal SUV (SUVmax) of the tumor was 3.24 (Fig. 2B). To evaluate the metabolic activity more objectively, relative SUV (RUV) was calculated by dividing the SUVmax of the adrenal tumor by the mean SUV (SUVmean) of the normal liver (Fig. 2D), and an RUV ratio of 5.23 was estimated. There was no evidence of metastatic lesion in the whole body, including liver, spleen, lung, lymph nodes, and regional lymph nodes based on the whole-body PET/CT images.

**Fig. 3:**
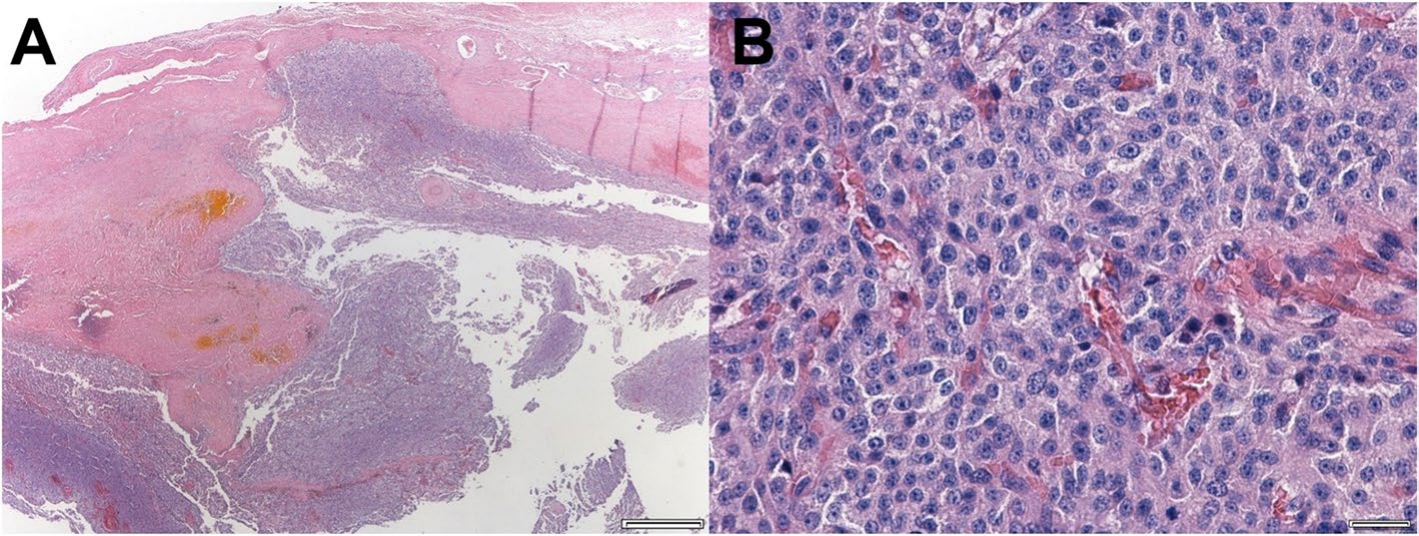
Histopathology of a right adrenal mass after adrenalectomy shows the characteristics of an adrenocortical carcinoma. **a** Image showing adrenal gland with capsular invasion of tumor cells and multiple area of hemorrhage (H&E stain, original magnification × 10. scale bar 500 μm) **b** Magnified image of the tumor presents neoplastic cells arranged in nesting pattern (H&E stain, original magnification × 400. scale bar 20 μm)

After the additional administration of prazosin 0.5 mg/kg/q12h for 1 month to relieve hypertension, surgical adrenalectomy was performed, and the adrenal mass was removed. Histopathologic examination of the resected adrenal mass revealed the characteristics of an adrenocortical carcinoma (Fig. 3). The architecture of the adrenal tissue was completely replaced by neoplastic cells, resulting in the loss of normal structures. Capsular invasion of tumor cells, multiple areas of necrosis and hemorrhage in the connective tissue with hematoidin crystals were detected. Pleomorphic tumor cells were subdivided by thin fibrous connective tissue, resulting in the formation of nests. They had abundant eosinophilic cytoplasm with some lipid vacuoles and round nuclei with prominent nucleoli. Mitotic index was 1–2 in all high-power fields. The histopathological slides were examined using Olympus SLIDEVIEW VS200 microscope and images were achieved using the Olympus OlyVIA software (Olympus). The dog was re-examined 2 weeks after the excision of the adrenal tumor, and the systolic blood pressure had reduced to below 150 mmHg without anti-hypertensive medication. The owners declined any further assessment, and the dog died after 31 months.

## Discussion and conclusions

In the present case, a hypertensive dog was suspected to have a malignant adrenocortical tumor based on the 18F-FDG/CT imaging, and histological examination was consistent with adrenocortical carcinoma.

Some adrenal tumors release excessive hormone produced by the adrenal gland, regardless of whether they originate from the cortex or medulla, or whether they are benign or malignant [16]. Pheochromocytomas arising from chromaffin cells of the adrenal medulla secrete catecholamine, and some adrenocortical tumors produce excessive amounts of cortisol. Several clinical signs of pheochromocytoma and cortisol-secreting tumors overlap, such as weakness, tachypnea, panting, polydipsia/polyuria, and hypertension, which makes it difficult to distinguish between the two tumors based only on their clinical characteristics [13, 17]. However, differentiation of both tumors is crucial because pre-operative medical management with phenoxybenzamine would be beneficial to minimize potentially life-threatening complications during surgery in dogs with pheochromocytoma [5]. In the present case, due to the time-consuming nature of LDDST and concerns about the risk of hypertensive crisis that might be caused by administration of dexamethasone [18, 19], the owners declined LDDST, and ACTH stimulation test was administered instead. Considering the results of the ACTH stimulation test with significantly increased concentrations of 17-OHP, estradiol, and progesterone in both before and after ACTH stimulation test, atypical hypercortisolism was suspected [20–22]; however, hypercortisolism was not confirmed on the basis of these results alone because of absence of LDDST. Furthermore, additional tests to identify pheochromocytoma were required, even if hypercortisolism existed, because pheochromocytoma and cortisol producing tumors can appear simultaneously in the same dog [23]. The urinary normetanephrine-to-creatinine ratio was 247.56 in this dog, and unfortunately, it was too low to diagnose pheochromocytoma and too high to rule out the possibility of pheochromocytoma. According to two previous studies, some dogs with hypercortisolism without pheochromocytoma (6 of 24, 25%) showed values more than 247.56, and some dogs with pheochromocytoma (3 of 14, 21.4%) showed values below 247.56 [13, 16]. Only the thin surface of the adrenal mass showed significantly increased 18F-FDG uptake on PET/CT, and the central region of the mass exhibited an absence of 18F-FDG uptake, which is known as a specific finding for necrosis in humans [24, 25]. These findings were highly suggestive of adrenocortical tumor rather than medullary tumor, although the diagnosis could not be made by uptake characteristics of 18F-FDG alone. Histopathologic evaluation revealed the characteristics of an adrenocortical carcinoma, and it consistent with the PET/CT examination.

Except for histologic examination, malignancy of adrenal tumor is challenging to identify using other various methods in veterinary medicine. Although cytology is useful in differentiating cortical-originated tumors from medullary-originated tumors, its application is limited for the evaluation of malignancy [26, 27]. Imaging modalities can be helpful in judging malignancy before surgical adrenalectomy. Abdominal ultrasound examination has been reported to sensitive and specific for the detection of invasion of adrenal tumor and metastasis [28], but many dogs with malignant adrenal tumors may not exhibit invasive or metastatic findings; only 20 and 50% of adrenocortical carcinomas have been reported with invasion and metastasis, respectively [29]. No evidence of invasion or metastasis was confirmed by 18F-FDG PET/CT in this case. Contrast-enhanced CT enables detection of vascular invasion and some CT findings are associated with pathologic characteristics [30]. However, there are overlapping features between tumor types, so distinguishing them based on CT alone is limited [30]. In a previous study, there was moderate agreement between the peripheral contrast-enhancing rim and the absence of capsular invasion on histological examination (kappa = 0.53, *P* = 0.05) [31]. In the present case, contrast enhanced peripheral region of the adrenal mass revealed capsular invasion on histological examination. Increased 18F-FDG uptake in the peripheral region suggests infiltration of neoplastic cells and it corresponds with histopathological findings.

In humans, 18F-FDG PET/CT has been used to differentiate between benign and malignant adrenal tumors. Adrenocortical carcinoma, which is an aggressive malignant tumor, usually leads to 18F-FDG accumulation within the lesion [31], while the majority of adenomas (common benign tumors) are hypometabolic [32]. To evaluate the 18F-FDG uptake of an adrenal tumor, two main methods are widely used: 1) 18F-FDG uptake of the lesion has been compared with that of the normal liver on visual evaluation, and 2) semiquantitative assessment has been performed: SUVmax and RUV (adrenal SUVmax/liver SUVmean) [10, 32, 33]. On visual interpretation, majority of lesions reported as showing higher 18F-FDG uptake than that of the normal liver, have been revealed as a malignancy; except for 3% of benign adrenal lesions that appeared with higher 18F-FDG uptake than that of the normal liver in humans [10, 33, 34]. Some studies have reported that an SUVmax from 2.3 to 3.1 demonstrated a sensitivity and specificity of around 100% and ranging from 78.1 to 94%, respectively, for a differentiation between benign and malignant adrenal lesions [10, 32]. In a previous study, sensitivity and specificity with the use of RUV values to differentiate adenomas from non-adenomas were 96, and 58%, respectively, using a cut-off < 1.5 [33]. In the present case, intensity of uptake of the adrenal mass was higher than that of the liver, with SUVmax 3.24 and RUV 5.23, and these values were consistent with carcinomas rather than adenomas according to previous reports in humans [10, 32, 33], although there were no reports in dogs. Therefore, it was possible to predict malignancy of the adrenal mass using 18F-FDG PET/CT, which could not be distinguished by ultrasound examinations and cytology.

In a study about the physiologic uptake of 18F-FDG in normal dogs, mean and maximum SUVs of the adrenal gland were approximately 0.89 (95% confidence intervals, 0.68–1.10), and 1.04 (95% CI, 0.71–1.36), respectively [35]. In this dog, SUVmean of the adrenal mass was 0.36, lower than the values in normal dogs because ROIs included a hypometabolic necrotic region that accounted for a large portion of the mass. Thus, SUVmean of the peripheral region of the adrenal mass assumed to be adrenal cortex was measured and was identified as 0.79 (Fig. 2C), similar to the values of normal dogs. Maximum SUVs of the adrenal mass was 3.24, significantly higher than that of normal dogs. Due to lack of PET/CT data in veterinary medicine, we could compare the 18F-FDG uptake in adrenal mass with values in only one study. Therefore, caution should be exercised when using above values to directly compare the SUV measured between different institutions.

18F-FDG accumulation provides important information useful for predicting the prognosis of the human patients with adrenocortical carcinoma [31]. The criteria for 18F-FDG uptake, SUVmax 10, has been proposed to indicate a poor prognosis in humans with adrenocortical carcinoma; 57% of the patients with lesion SUVmax > 10 died within 6 months, but none of the patients with lesion SUVmax ≤10 died [31]. In the present case, the maximum SUVs were less than 10, (SUVmax = 3.24), which was not considered high, considering the prior study in humans [31], and the dog survived for more than 6 months. This dog died 31 months after the adrenalectomy.

A diagnosis of adrenocortical tumor can be made without special stains. However, considering the present dog’s clinical presentation and urine normetanephrine results, suspicion for pheochromocytoma would still exist because adrenal gland tumors can be mixed and the histopathologic appearance can be affected upon sectioning. Unfortunately, special staining procedures for ruling out or confirming concurrent pheochromocytoma were not performed for this case. Therefore, it was not possible to rule out a possibility of concurrent pheochromocytoma because immunohistochemistry for chromogranin A, a neuroendocrine marker for pheochromocytoma [1], was not performed. Ki-67 immunostaining that is useful to differentiate benign and malignant adrenal tumors [36] was not also performed although > 1 mitosis per high-power fields were highly specific for adrenocortical carcinoma [36] and other histopathological findings were consistent with those of carcinoma in the present case. Similarities in clinical features of pheochromocytomas and hypercortisolism make the differentiation quite challenging [37], and a dog having atypical hyperadrenocorticism with concurrent pheochromocytoma was reported, although histopathologic confirmation was not performed [38]. Moreover, ectopic ACTH secretion from undetected pheochromocytoma, triggering atypical hyperadrenocorticism could have occurred. Therefore, the clinical features of the present case could be due to adrenocortical carcinoma, pheochromocytoma, or both. The 18F-FDG PET/CT characteristics and the result of routine histopathologic examination might be consistent with adrenocortical carcinoma in the present case, but further evaluation using immunohistochemical analyses using chromogranin A and Ki-67 will be necessary to confirm it in dogs similar to those seen in the present case.

This is the first case report of 18F-FDG PET/CT findings in a dog with suspected adrenocortical carcinoma. In the present case, because the peripheral region of the adrenal mass revealed significantly increased 18F-FDG uptake, but not the central region, an adrenocortical malignant tumor was strongly suspected, and it was confirmed with histopathology following adrenalectomy. This case may provide valuable diagnostic information for adrenocortical carcinomas in dogs, and further studies are needed to establish diagnostic criteria for the PET/CT examination of canine adrenal tumors.

## Data Availability

Relevant data are fully within this paper. The datasets are available from the corresponding author on reasonable request.

## Abbreviations

ACTH: Adrenocorticotrophic hormone
CT: Computed tomography
FDG: 18F-fluorodeoxyglucose
LDDST: Low dose dexamethasone suppression test
OHP: Hydroxyprogesterone
PET: Positron emission tomography
RI: Reference interval
ROI: Region of interest
RUV: Relative standardized uptake value
SUV: Standardized uptake value
SUVmax: Maximal standardized uptake value
SUVmean: Mean standardized uptake value
